# The cell surface hyaluronidase TMEM2 is essential for systemic hyaluronan catabolism and turnover

**DOI:** 10.1016/j.jbc.2021.101281

**Published:** 2021-10-06

**Authors:** Yuki Tobisawa, Naoki Fujita, Hayato Yamamoto, Chikara Ohyama, Fumitoshi Irie, Yu Yamaguchi

**Affiliations:** 1 Human Genetics Program, Sanford Burnham Prebys Medical Discovery Institute, La Jolla, California, USA; 2 Department of Urology, Hirosaki University Graduate School of Medicine, Hirosaki, Japan

**Keywords:** TMEM2, hyaluronan, hyaluronidase, gene knockout, carbohydrate metabolism, lymph node, liver, endothelial cell, HA, hyaluronan, FA-HA, fluoresceinamine-labeled hyaluronan, IVC, inferior vena cava, ISH, *in situ* hybridization, PBS, phosphate-buffered saline, HMW, high molecular weight, HABP, hyaluronan binding protein

## Abstract

As a major component of the extracellular matrix, hyaluronan (HA) plays an important role in defining the biochemical and biophysical properties of tissues. In light of the extremely rapid turnover of HA and the impact of this turnover on HA biology, elucidating the molecular mechanisms underlying HA catabolism is key to understanding the *in vivo* functions of this unique polysaccharide. Here, we show that TMEM2, a recently identified cell surface hyaluronidase, plays an essential role in systemic HA turnover. Employing induced global *Tmem2* knockout mice (*Tmem2*^*iKO*^), we determined the effects of *Tmem2* ablation not only on the accumulation of HA in bodily fluids and organs, but also on the process of HA degradation *in vivo*. Within 3 weeks of tamoxifen-induced *Tmem2* ablation, *Tmem2*^*iKO*^ mice exhibit pronounced accumulation of HA in circulating blood and various organs, reaching levels as high as 40-fold above levels observed in control mice. Experiments using lymphatic and vascular injection of fluorescent HA tracers demonstrate that ongoing HA degradation in the lymphatic system and the liver is significantly impaired in *Tmem2*^*iKO*^ mice. We also show that *Tmem2* is strongly expressed in endothelial cells in the subcapsular sinus of lymph nodes and in the liver sinusoid, two primary sites implicated in systemic HA turnover. Our results establish TMEM2 as a physiologically relevant hyaluronidase with an essential role in systemic HA catabolism *in vivo*, acting primarily on the surface of endothelial cells in the lymph nodes and liver.

Hyaluronan (HA), a member of the family of glycosaminoglycans (GAGs), is a long unbranched polysaccharide (as along as 25,000 disaccharide units in length) with a molecular weight reaching as high as 10^7^ Da. HA is a major component of the extracellular matrix (ECM) and is also present in biological fluids, such as blood and lymph (1, 2, 3). HA is extremely hydrophilic and occupies a large hydrodynamic volume in solution. These unique biochemical and biophysical properties of HA are accompanied and influenced by another unique feature of HA metabolism—an extremely rapid turnover. An estimated one-third of total body HA (∼15 g in a person with a 70 kg body weight) is turned over daily (4), and the metabolic half-life of HA in skin is only 1–1.5 days (5). This is in stark contrast to half-lives of other ECM molecules in tissues, many of which have half-lives measured in months or years (6, 7, 8). This rapid turnover suggests that cells must possess extremely robust machinery for HA degradation.

Degradation of HA is thought to be a multistep process. The widely accepted, although not thoroughly tested, model proposes that HA in the extracellular space is first partially degraded into intermediate-sized HA fragments by an extracellular or cell surface hyaluronidase (9). Subsequently, these intermediate-sized HA species are internalized and further degraded by hyaluronidase(s) in endosomes and lysosomes. These smaller HA fragments are ultimately degraded by lysosomal exoglucosidases into monosaccharides, which are then recycled for the production of new glycans or for glycolysis (1). On the systemic level, these stages of HA degradation do not necessarily occur in a single cell type. While some of the HA released from the ECM of peripheral tissues is taken up and metabolized by local cells, the bulk of released HA flows into lymphatic vessels and is transported into the lymphatic nodes, where the majority of degradation occurs (10). In addition, HA present in blood plasma is primarily taken up and degraded by the liver (4).

Five major proteins are known to function in HA degradation in mammalian tissues, namely HYAL1, HYAL2, SPAM1 (also known as PH-20), HYBID (Hyaluronan Binding Protein Involved in Hyaluronan Depolymerization; also known as KIAA1199 and CEMIP), and TMEM2 (Transmembrane protein 2; also known as CEMIP2) (11). Sharing high levels of sequence homology, HYAL1, HYAL2, and SPAM1 constitute a protein family whose members are thought to have been generated by gene duplication (12). SPAM1, the first identified member of the family, is predominantly expressed in the testis (13). HYAL1 was originally purified from human plasma as a 57 kDa protein (14), while HYAL2 was identified by cDNA cloning based on sequence homology with SPAM1 (15). There has been a debate concerning the subcellular localization of the HYAL proteins. While HYAL2 was originally identified in lysosomes of glioma cells (15) and is present in the lysosomes of other cell types (16, 17), it has also been shown to be anchored on the cell surface *via* a glycosylphosphatidylinositol (GPI) linkage (18, 19). While the bulk of SPAM1 is present in the acrosome, a lysosome-related organelle of sperm cells (20), SPAM1 can also be anchored on the cell surface *via* a GPI linkage (13). The pH optima for the hyaluronidase activities of HYAL1 and HYAL2 are both distinctly acidic (15, 21), consistent with the possibility that they function primarily in the lysosome. SPAM1 exhibits dual pH optima at acidic and neutral ranges (22). HYAL family proteins degrade not only HA but also chondroitin sulfate and dermatan sulfate (9, 23). HYBID and TMEM2 constitute a family of proteins independent of the HYAL family. HYBID was originally identified as a secretory protein that has HA binding activity (24). Transfection with HYBID confers HEK293 cells with the ability to degrade HA, while knockdown of HYBID inhibits the endogenous HA degrading activity of Detroit 511 fibroblasts (24), indicating that HYBID plays a role in HA degradation. Curiously, however, neither conditioned media of HYBID-transfected cells nor recombinant HYBID protein exhibits intrinsic HA degrading activity (24, 25). Instead, HYBID-mediated HA degradation requires participation of the clathrin-coated pit pathway (24), suggesting that HYBID itself might not be a hyaluronidase *per se*.

TMEM2 was originally identified as one of more than 300 different human open reading frames that are grouped together based only on the presence of at least one putative transmembrane domain (26). The observations that TMEM2 has sequence similarity with HYBID, and that zebrafish *tmem2* mutants exhibit excessive HA accumulation in developing heart (27), suggested to us that TMEM2 might be an unidentified cell surface hyaluronidase. Subsequently, we demonstrated that TMEM2 is a type II transmembrane protein that has intrinsic hyaluronidase activity at neutral pH (28). More recently, we showed that endogenous TMEM2 degrades substrate-immobilized HA in a contact-dependent manner, further supporting the notion that it acts as a cell surface hyaluronidase (29). TMEM2 is expressed widely in adult mouse tissues, including the lymph nodes and liver (28), the tissues primarily implicated in systemic HA catabolism.

In this study, we use conditional *Tmem2* knockout mice to investigate the functional significance of TMEM2 in systemic HA catabolism. Seeking to define a specific role for TMEM2 in physiological HA catabolism, we have induced global *Tmem2* ablation in adult mice. These knockout mice have allowed us to demonstrate a rapid accumulation of HA in bodily fluids and organs during a short window of time following the induction of *Tmem2* ablation, as well as deleterious effects of *Tmem2* ablation on ongoing degradation of HA in the vascular and lymphatic systems. Moreover, we show that TMEM2 is expressed in the endothelial cells in the lymph nodes and liver, two predominant sites implicated in systemic HA turnover. Overall, our results establish that TMEM2 is a physiological hyaluronidase that plays an essential role in systemic HA catabolism *in vivo*.

## Results

### Induced global ablation of Tmem2 leads to rapid accumulation of HA in bodily fluids and in a variety of organs

**Figure S1:**
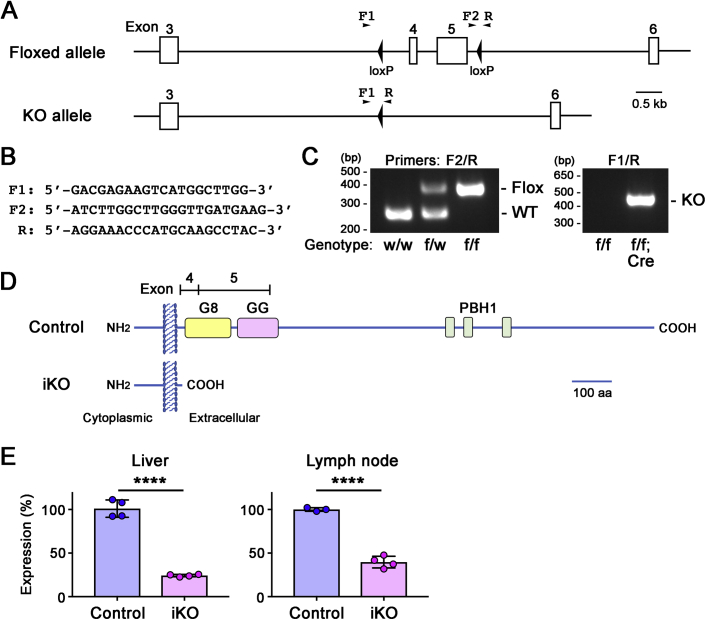
**Creation of a conditional *Tmem2***^***flox***^**allele and generation of induced global *Tmem2* KO (*Tmem2***^***iKO***^**) mice.***A*, schematic diagram of the *Tmem2*^*flox*^ allele before (*top*) and after (*bottom*) Cre-mediated recombination. Exons 4 and 5 are flanked with loxP sites. Locations of PCR primers for genotyping (F1, F2, R) are shown by *arrowheads*. *B*, sequence of PCR primers shown in *panel A*. *C*, representative results of genotyping PCR. Template genomic DNA was prepared from tails of WT (*w/w*), *Tmem2*^*flox/wt*^ (*f/w*), *Tmem2*^*flox/flox*^ (*f/f*), and *Tmem2*^*iKO*^ (*f/f;Cre*; *i.e.*, *CAG-Cre*^*ER*^*;Tmem2*^*flox/flox*^) mice having undergone the tamoxifen induction regimen (see Experimental procedures). *D*, schematic diagram of TMEM2 proteins produced in WT (*top*) and *Tmem2*^*iKO*^ (*bottom*) mice. Cre-mediated recombination deletes the entire G8 domain, which contains several amino acid residues essential for the catalytic activity of TMEM2, as well as the bulk of the GG domain, and creates a frame shift that results in a premature stop codon just downstream of the transmembrane domain. *E*, reduction in *Tmem2* expression upon tamoxifen treatment in the liver and lymph nodes. The liver and lymph nodes were dissected from *Tmem2*^*iKO*^ and control mice 3 days after the final tamoxifen injection and used for isolation of total RNA from which cDNA was prepared. *Tmem2* expression in the liver and lymph node was determined by using TaqMan qPCR, and levels of *Tmem2* expression was calculated by the ΔΔCt method using normalization to ribosomal 18S RNA. Experiments were performed in biological triplicates or quadruplicates, and statistical significance was analyzed by Student's *t*-test. ∗∗∗∗ *p* < 0.0001.

To define the physiological significance of TMEM2 in systemic HA catabolism, we generated inducible *Tmem2* knockout mice using a floxed *Tmem2* allele (*Tmem2*^*flox*^; Fig. S1, *A–D*) and the *CAG-Cre*^*ER*^ transgene. *CAG-Cre*^*ER*^ is ubiquitously active, tamoxifen-inducible Cre driver (30). An important advantage of this inducible global knockout system is that it allows investigation of the effect of *Tmem2* ablation on current, ongoing systemic HA catabolism. In contrast, models using constitutive knockout of hyaluronidase genes to assess the consequence of loss of enzyme activity must inevitably reflect the long-term accumulation of HA in the tissues and blood, including even the embryonic and early postnatal periods of life. HA accumulation in constitutive knockout models could thus be a long-term, indirect consequence of gene ablation, rather than the direct consequence of loss of the targeted gene on current HA metabolism. Inducible gene ablation avoids these intrinsic shortcomings of constitutive knockout models and importantly, allows determination of the time course of HA accumulation shortly after the induction of gene ablation.

*CAG-Cre*^*ER*^*;Tmem2*^*flox/flox*^ mice at 6 weeks of age were treated with tamoxifen (0.125 mg/g body weight/day, *i.p*., for 5 consecutive days) to induce global *Tmem2* ablation (hereafter, these mice will be referred to as *Tmem2*^*iKO*^ mice). As controls, *Tmem2*^*flox/flox*^ mice were treated with tamoxifen in the same manner (hereafter, these mice will be referred to as control mice). Knockout efficiency in *Tmem2*^*iKO*^ mice as analyzed by TaqMan qPCR was 76% and 61% in the liver and lymph nodes, respectively (Fig. S1*E*). These levels of knockout efficiency are comparable to other values reported using the same *CAG-Cre*^*ER*^ system (31, 32, 33, 34, 35, 36, 37).

**Figure 1:**
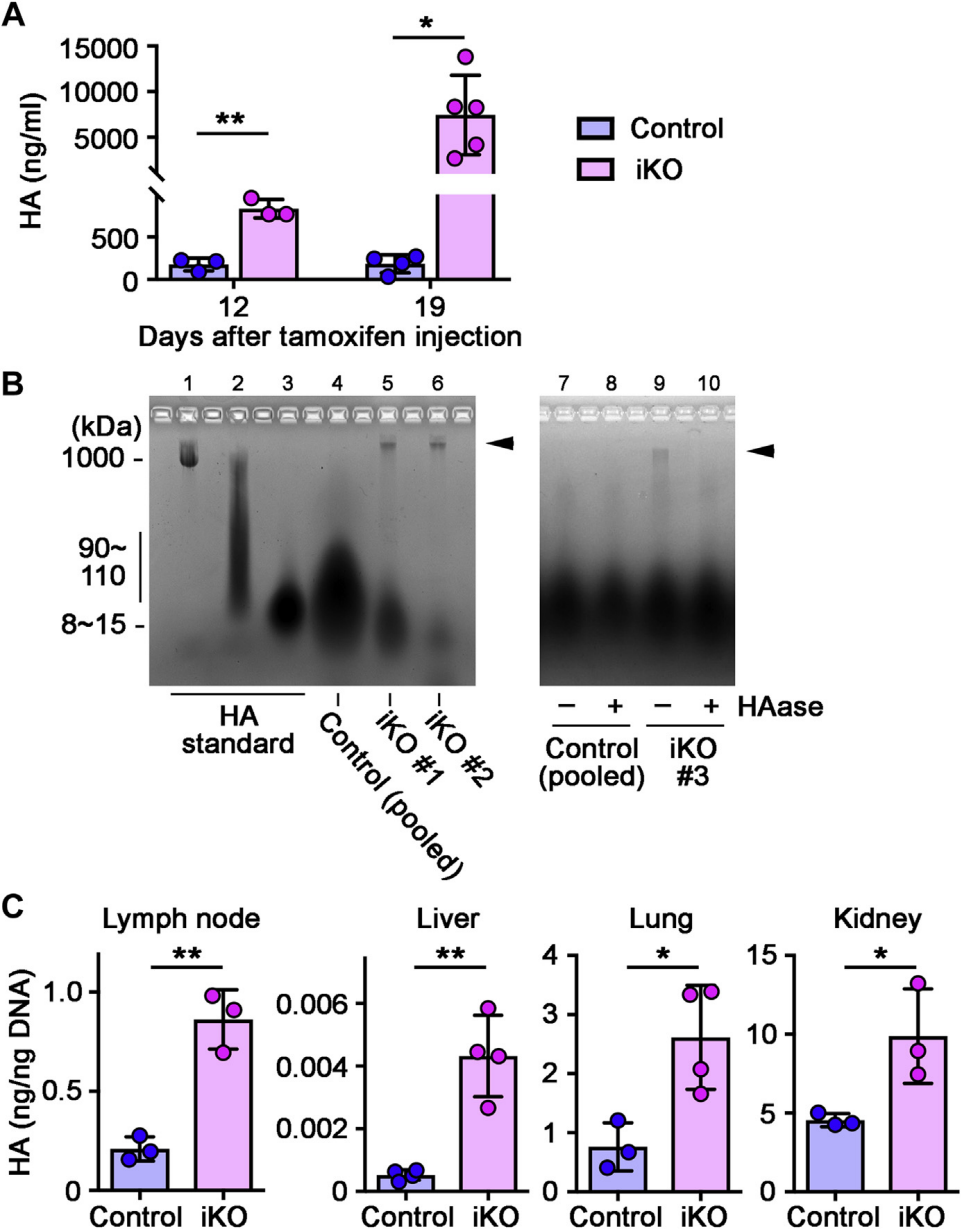
**Induced global ablation of *Tmem2* leads to rapid accumulation of HA in bodily fluid and organs.***A*, HA accumulation in circulating blood at day 12 and day 19 after the completion of tamoxifen treatment. Data represent means ± SD (n = 3–6 per genotype). ∗*p* < 0.05, ∗∗ *p* < 0.01 by Student's *t* test. *B*, Persistence of HMW HA in circulating blood of *Tmem2*^*iKO*^ mice. Plasma from *Tmem2*^*iKO*^ and control mice at day 19 was analyzed by agarose-gel electrophoresis and Stains-All staining. *Lanes 1–3*: HA molecular weight standards (from *left* to *right*: 1000 kDa, 90–110 kDa, 8–15 kDa). *Lane 4*: Total HA pooled from ten control mice. *Lanes 5 and 6*: Total HA from two independent *Tmem2*^*iKO*^ mice. Note that *lane 4* was loaded with excess amount of total HA in order to demonstrate that essentially no undigested HMW HA is present in blood of control mice. *Lanes 7–10*: Control experiments to show that the HMW bands in *lanes 5*, *6*, and *9* (indicated by *arrowheads*) are indeed HA. Plasma samples were pretreated with (+) or without (–) *Streptomyces* hyaluronidase (*HAase*) prior to electrophoresis. *C*, HA accumulation in the lymph nodes, liver, lung, and kidney at day 19 after the completion of tamoxifen treatment. Data represent means ± SD (n = 3–4 per genotype). ∗ *p* < 0.05, ∗∗ *p* < 0.01 by Student's *t* test.

At day 12 and day 19 after the treatment (the day of the final [fifth] tamoxifen injection is designated as day 0), mice were euthanized, and tissues and blood were collected for the analysis of HA. *Tmem2*^*iKO*^ mice exhibit rapid and massive accumulation of HA in blood. The blood HA level in *Tmem2*^*iKO*^ mice was 832 ± 63 ng/ml at day 12 and 7452 ± 1943 ng/ml at day 19, values that are 4.7 and 40.5 times higher than those seen in control mice at the respective time points (Fig. 1*A* and Table S1). Agarose-gel electrophoresis with the Stains-All dye (38) was used to further characterize the nature of HA in blood (Fig. 1*B*). This assay reveals that high molecular weight (HMW) bands of ∼1000 kDa (Fig. 1*B*, *arrowheads* in lanes 5 and 6) are present in *Tmem2*^*iKO*^ mice but not in control mice (lane 4). These HMW bands in *Tmem2*^*iKO*^ mice completely disappear following treatment with *Streptomyces* hyaluronidase (Fig. 1*B*, compare lanes 9 and 10), indicating that they indeed represent HA. Thus, in *Tmem2*^*iKO*^ mice, not only the HA content of circulating blood is markedly increased, but this circulating HA population contains a significant amount of undigested HMW HA species.

**Figure 2:**
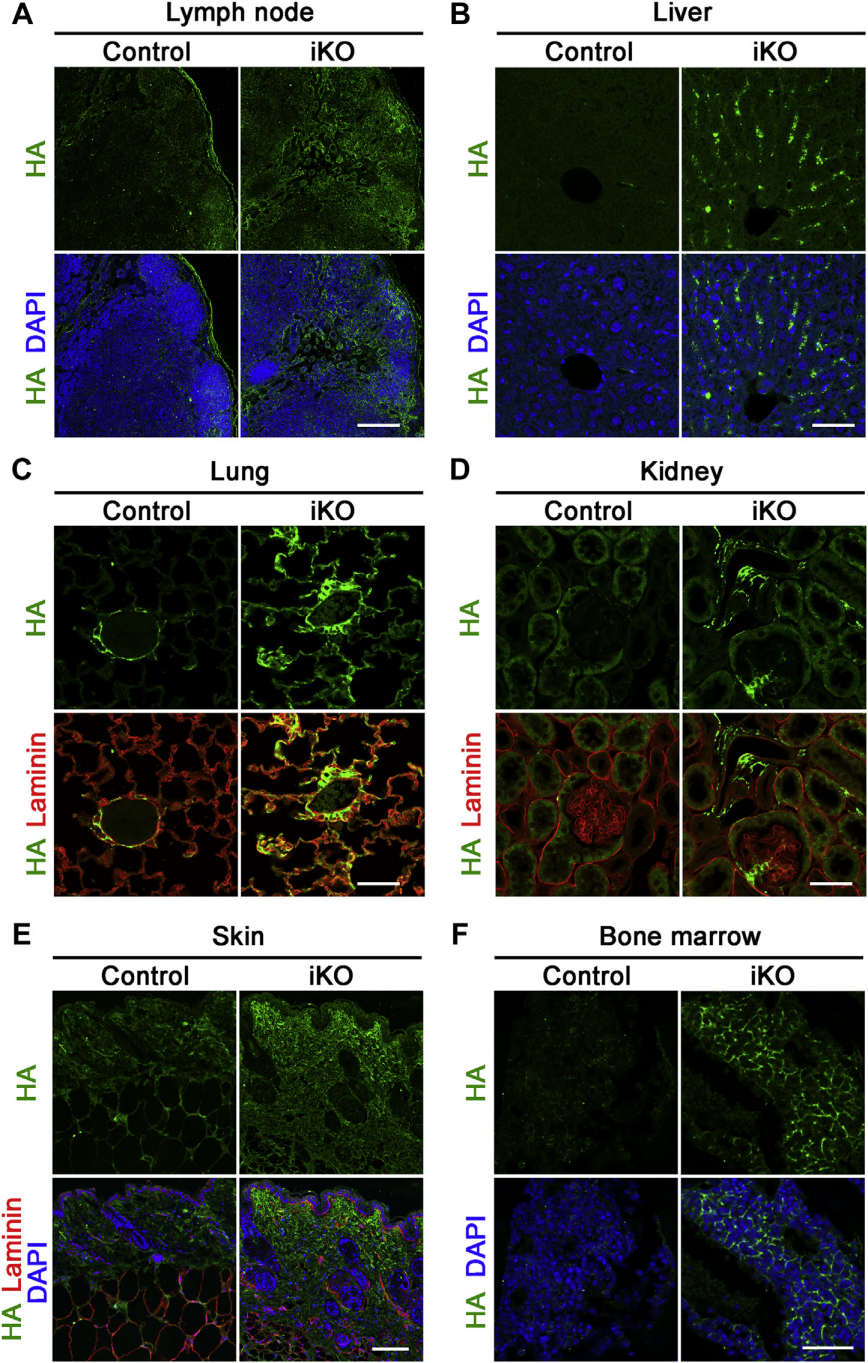
**Deposition of HA in tissues and organs of *Tmem2***^***iKO***^**mice.** Sections of the lymph nodes (*A*), liver (*B*), lung (*C*), kidney (*D*), skin (*E*), and bone marrow (*F*) at day 19 after the completion of tamoxifen treatment were stained with biotinylated HABP and AlexaFluor 488-conjugated streptavidin. Sections of the skin, lung, and kidney were also stained for laminin to better delineate tissue structures. Scale bars, 200 μm (*A*), 50 μm (*B*), 50 μm (*C*), 50 μm (*D*), 100 μm (*E*), 100 μm (*F*).

**Figure S2:**
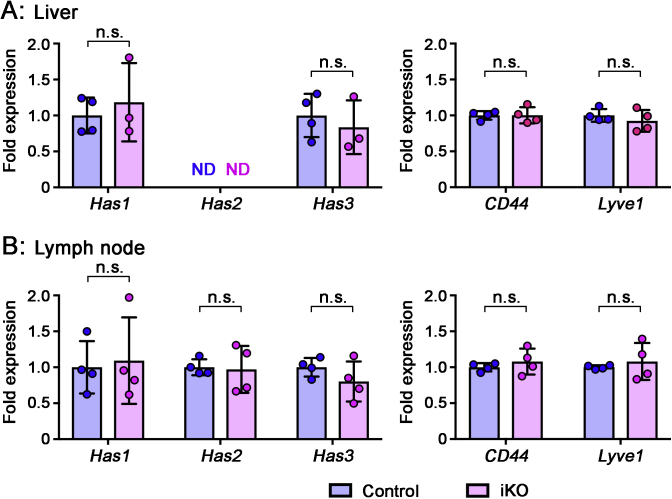
**Expression of HA synthases and major HA-binding proteins is not altered in *Tmem2***^***iKO***^**mice.** The liver and lymph nodes were dissected from control and *Tmem2*^*iKO*^ mice at day 19 after the completion of tamoxifen treatment, which were then used for total RNA isolation and cDNA synthesis. Expression levels of *Has1*, *Has2*, *Has3*, *CD44*, and *Lyve1* in the liver (*A*) and lymph node (*B*) were determined by TaqMan qPCR, and fold changes in expression levels were calculated by the ΔΔCt method using normalization to ribosomal 18S RNA. Experiments were performed in biological triplicates or quadruplicates. Data are shown as the mean ± SD, and statistical significance was analyzed by two-way ANOVA with Bonferroni's multiple comparison test. ND, not detected; n.s., not significant.

HA accumulation in tissues was examined by biochemical HA quantification and by tissue HA staining in *Tmem2*^*iKO*^ and control mice at day 19. The HA content in lymph nodes in *Tmem2*^*iKO*^ mice is 861 ± 86 pg/ng DNA at day 19, which is 4.1 times higher than the level in control mice (210 ± 35 pg/ng DNA) (Fig. 1*C* and Table S2). Likewise, tissue HA contents are increased by 8.3-, 3.4-, and 2.2-fold in the liver, lung, and kidney, respectively, in *Tmem2*^*iKO*^ mice (Fig. 1*C* and Table S2). Deposition of HA was examined by tissue staining with the biotinylated HABP (HA-binding protein) probe in the four organs mentioned above, as well as in the skin and bone marrow. All these organs in *Tmem2*^*iKO*^ mice exhibit varying degrees of increased HA deposition (Fig. 2). In lymph nodes of *Tmem2*^*iKO*^ mice, the stromal components, most notably the subcapsular sinus, exhibit prominent HA deposition (Fig. 2*A*). In the liver, HA deposition is observed along the liver sinusoid (Fig. 2*B*). In other organs and tissues examined, HA deposition is prominent in the alveolar wall of the lung (Fig. 2*C*), the mesangium of the kidney (Fig. 2*D*), the dermis (Fig. 2*E*), and the bone marrow matrix (Fig. 2*F*). These increases in HA in blood and tissues are not due to enhanced expression of HA synthases in *Tmem2*^*iKO*^ mice, as qPCR analyses reveal no differences in their expression levels between *Tmem2*^*iKO*^ and control mice (Fig. S2). Expression of two major HA-binding proteins, namely CD44 and Lyve1, was not altered in *Tmem2*^*iKO*^ mice, either.

### TMEM2 is expressed in endothelial cells of the lymph nodes and liver, two organs implicated in systemic HA turnover

**Figure 3:**
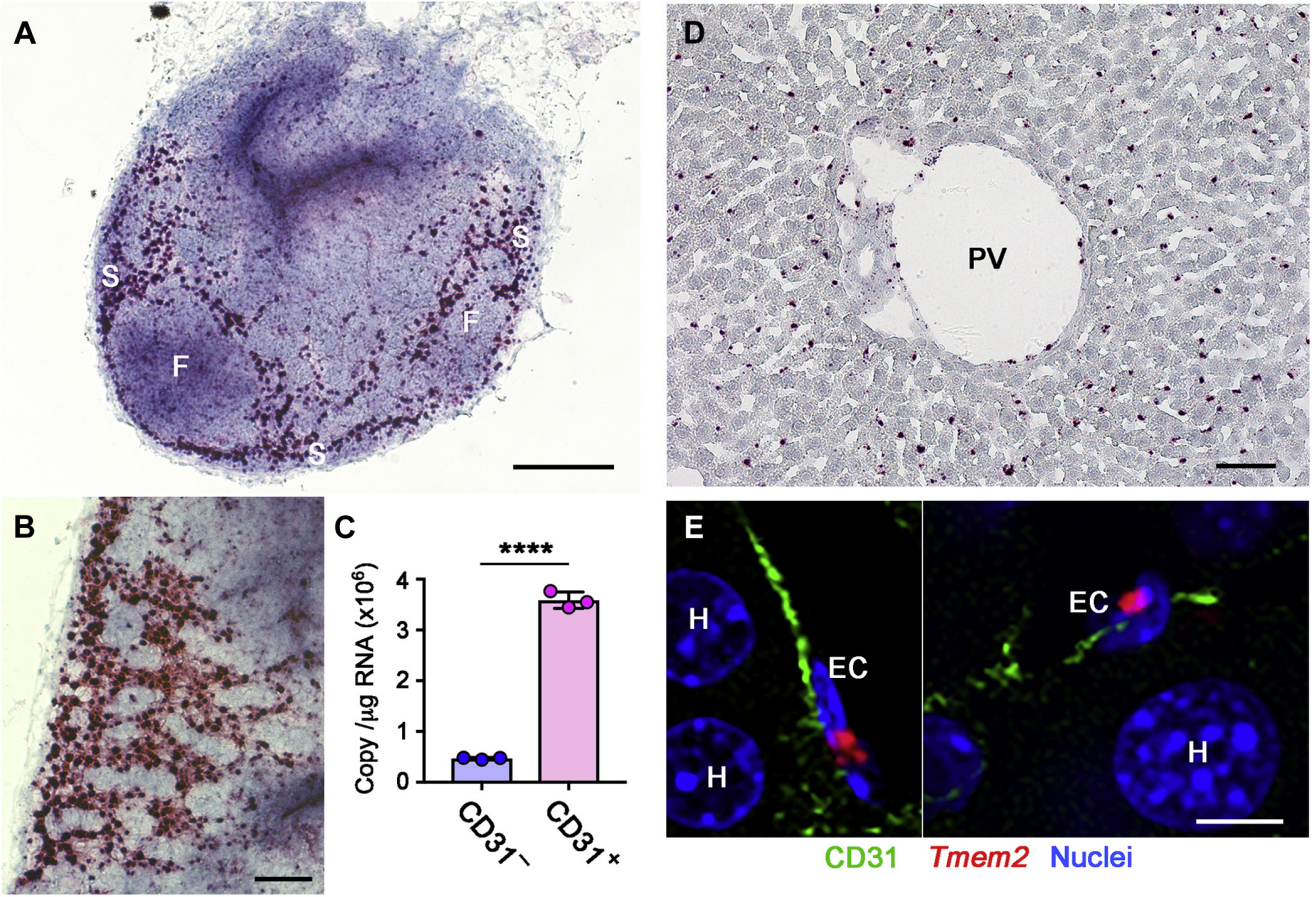
***Tmem2* expression in the lymph nodes and liver.***A* and *B*, *Tmem2* expression in lymph nodes. RNAscope ISH was performed on sections of inguinal lymph nodes from wild-type mice. Panel B shows a high power view of the subcapsular sinus. S, subcapsular sinus; F, follicle. Scale bars, 250 μm (*A*); 100 μm (*B*). *C*, *Tmem2* expression in CD31-positive endothelial cells isolated from lymph nodes. CD31-positive (*CD31*^*+*^) and CD31-negative (*CD31*^*–*^) cell populations were isolated by magnetic sorting and copy numbers of *Tmem2* transcripts were quantified by absolute RT-PCR. Data represent mean ± SD (n = 3 per cell population). ∗∗∗∗ *p* < 0.0001 by Student's *t* test. *D*, *Tmem2* expression in the liver. RNAscope ISH was performed on sections of the liver from wild-type C57Bl/6 mice. *PV*, portal vein. Scale bar, 50 μm. *E*, *Tmem2* expression in endothelial cells lining the liver sinusoids. Triple labeling by RNAscope ISH for *Tmem2* (*red*), immunohistochemistry for CD31 (*green*), and DAPI staining (*blue*) were performed on the liver sections from wild-type mice. *EC*, endothelial cell; *H*, hepatocyte (nucleus). Scale bar, 10 μm.

Lymph nodes and liver have been implicated as key sites in systemic HA turnover (1, 10). The rapid accumulation of HA in blood and lymph nodes observed in *Tmem2*^*iKO*^ mice is consistent with the notion that TMEM2 plays a physiologically relevant role in systemic HA catabolism. We therefore used the RNAscope *in situ* hybridization (ISH) technique to examine *Tmem2* mRNA expression patterns in the lymph nodes and liver. In lymph node of wild-type mice, strong *Tmem2* expression is detected primarily at sites corresponding to stromal components (Fig. 3, *A* and *B*). *Tmem2* expression is particularly robust in cells of the subcapsular sinus (*S*), whereas signals are negligible in the lymphoid follicles (*F*). Since lymphatic endothelial cells have been implicated as the cell type primarily responsible for HA degradation in the lymphatic system (10, 39), we used magnetic cell sorting to isolate CD31-positive endothelial cells and CD31-negative nonendothelial cells from lymph nodes and analyzed *Tmem2* expression by absolute quantification RT-PCR. As shown in Figure 3*C*, CD31-positive cells express *Tmem2* much more strongly than CD31-negative cells. Together with the RNAscope ISH results, these results demonstrate that endothelial cells in lymph nodes, in particular those in the subcapsular sinus, are the primary site of *Tmem2* expression.

In the liver, *Tmem2* mRNA is detected in cells that are regularly aligned along the liver sinusoid, while hepatocytes exhibit no *Tmem2* expression (Fig. 3*D*). This pattern of mRNA distribution suggests that *Tmem2* is expressed in the liver sinusoidal endothelial cells. This is consistent with a single-cell RNAseq data set (40) deposited in the Single Cell Portal database (Broad Institute; Study title: *Single-cell transcriptomics reveals early emergence of liver parenchymal and nonparenchymal cell lineages*), which demonstrates preferential *Tmem2* expression in endothelial cells. To further confirm *Tmem2* expression in liver sinusoidal endothelial cells, we performed an RNAscope ISH/CD31 immunohistochemistry double-labeling experiment. Results of this analysis show that *Tmem2* mRNA signals are associated with CD31-positive endothelial cells lining the liver sinusoids (Fig. 3*E*).

### Role of TMEM2 in dynamic HA degradation in the lymphatic system

**Figure 4:**
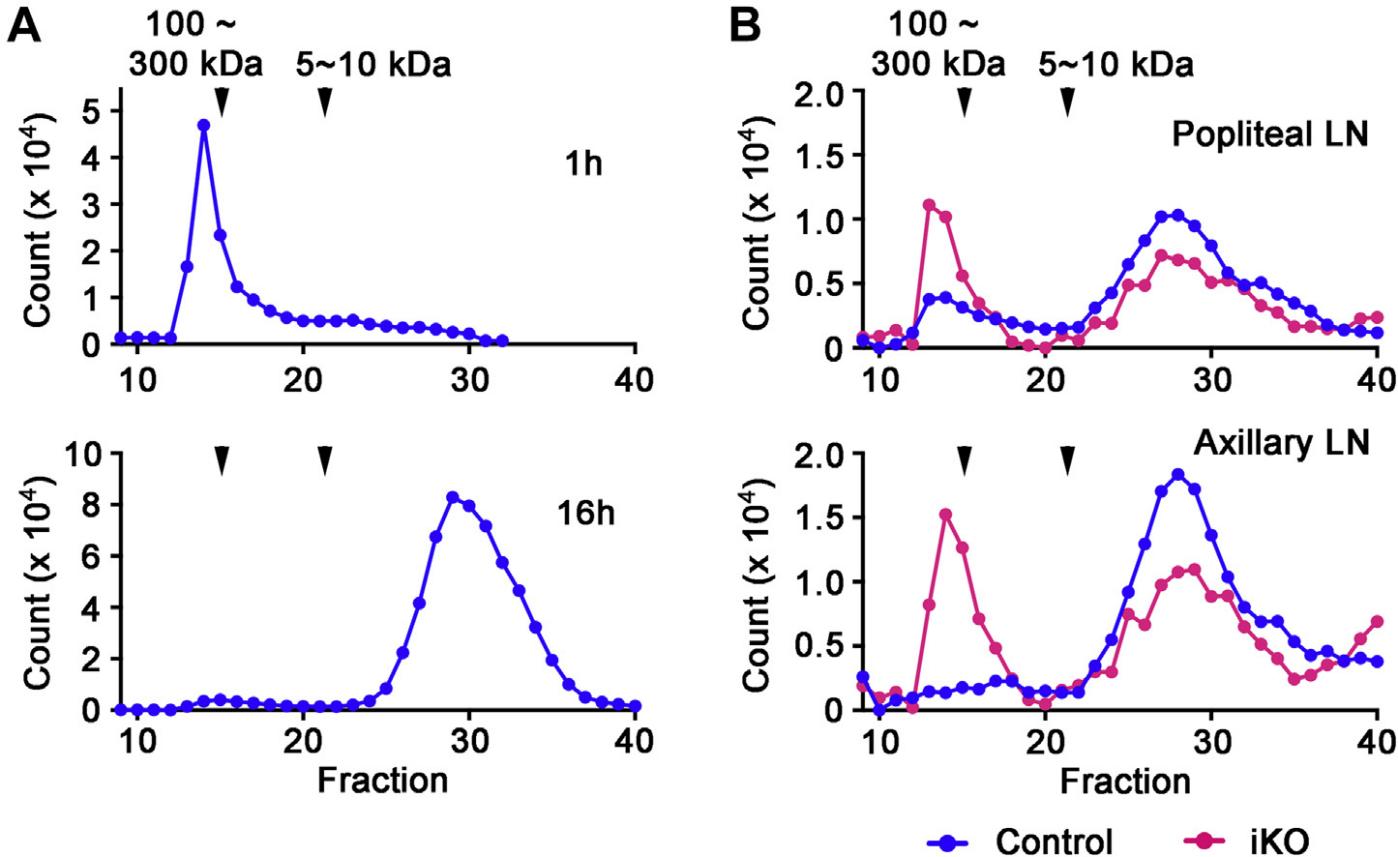
**Role of TMEM2 in dynamic HA degradation in the lymphatic system**. *A*, time course analysis of lymphatic HA degradation in wild-type mice. FA-HA (100∼300 kDa) was injected subcutaneously into the left hindlimb paw of adult wild-type mice, and the degradation pattern of FA-HA in the popliteal lymph node at 1 and 6 h after the injection was analyzed by Sephacryl S-300 chromatography. *B*, degradation patterns of FA-HA analyzed by gel filtration on Sephacryl 300 HR. *Upper panel*, analysis of hindlimb-injected FA-HA in the popliteal lymph nodes; *lower panel*, analysis of forelimb-injected FA-HA in the axillary lymph nodes. Elution profiles were normalized to the total fluorescence counts eluted from the column. *Tmem2*^*iKO*^ (*red*); control (*blue*).

Our results demonstrating HA accumulation in the wake of *Tmem2* ablation strongly support the physiological significance of TMEM2 in systemic HA catabolism. Nevertheless, the accumulation of HA is an indirect measure of HA catabolism and does not directly establish the physiological significance of a given hyaluronidase molecule in the *ongoing* process of HA degradation. Therefore, we employed *in vivo* injection of fluoresceinamine-labeled HA (FA-HA) (41) to define the effect of *Tmem2* ablation on the short-term dynamics of HA degradation. First, we examined the time course of lymphatic degradation of FA-HA injected into the peripheral tissue of wild-type mice. Right hindlimb paws of 10-week-old wild-type C57Bl/6 mice were injected with 15 μl of FA-HA (100–300 kDa, 1 μg/μl in PBS). At 1 or 16 h after injection, the popliteal lymph nodes were removed for analysis of HA degradation by gel filtration of tissue extracts. This analysis demonstrates that there is little degradation of FA-HA after 1 h (Fig. 4*A*, upper panel), but that FA-HA is almost completely degraded at 16 h (Fig. 4*A*, lower panel). To minimize potential confounding effects due to the longer postinjection interval, we chose a 6 h postinjection interval for subsequent experiments in *Tmem2*^*iKO*^ and control mice. In control mice, the majority of FA-HA species recovered from the popliteal lymph nodes at 6 h postinjection are small fragments of less than 10 kDa (Fig. 4*B*, upper panel, *blue*). In contrast, in *Tmem2*^*iKO*^ mice, the fragmentation of HA is significantly reduced; about half of the HA species recovered from the popliteal lymph nodes represent undigested HA (Fig. 4*B*, upper panel, *red*). We performed the same experiment for the forelimb lymphatics, injecting FA-HA into forelimb paws and removing the axillary lymph nodes at 6 h. This experiment yields results similar to those obtained for hindlimb injections (Fig. 4*B*, lower panel). These results demonstrate that the TMEM2 protein plays a physiological role in ongoing HA degradation in the peripheral lymphatic system. Although our experiments do not distinguish whether degradation occurs within the lymphatic vessels or in lymph nodes, the high density of *Tmem2* mRNA in lymphatic endothelial cells in the subcapsular sinus (see Fig. 3, *A* and *B*) strongly suggests that the bulk of HA degradation occurs in lymph nodes.

### Role of TMEM2 in dynamic HA degradation in the liver

**Figure 5:**
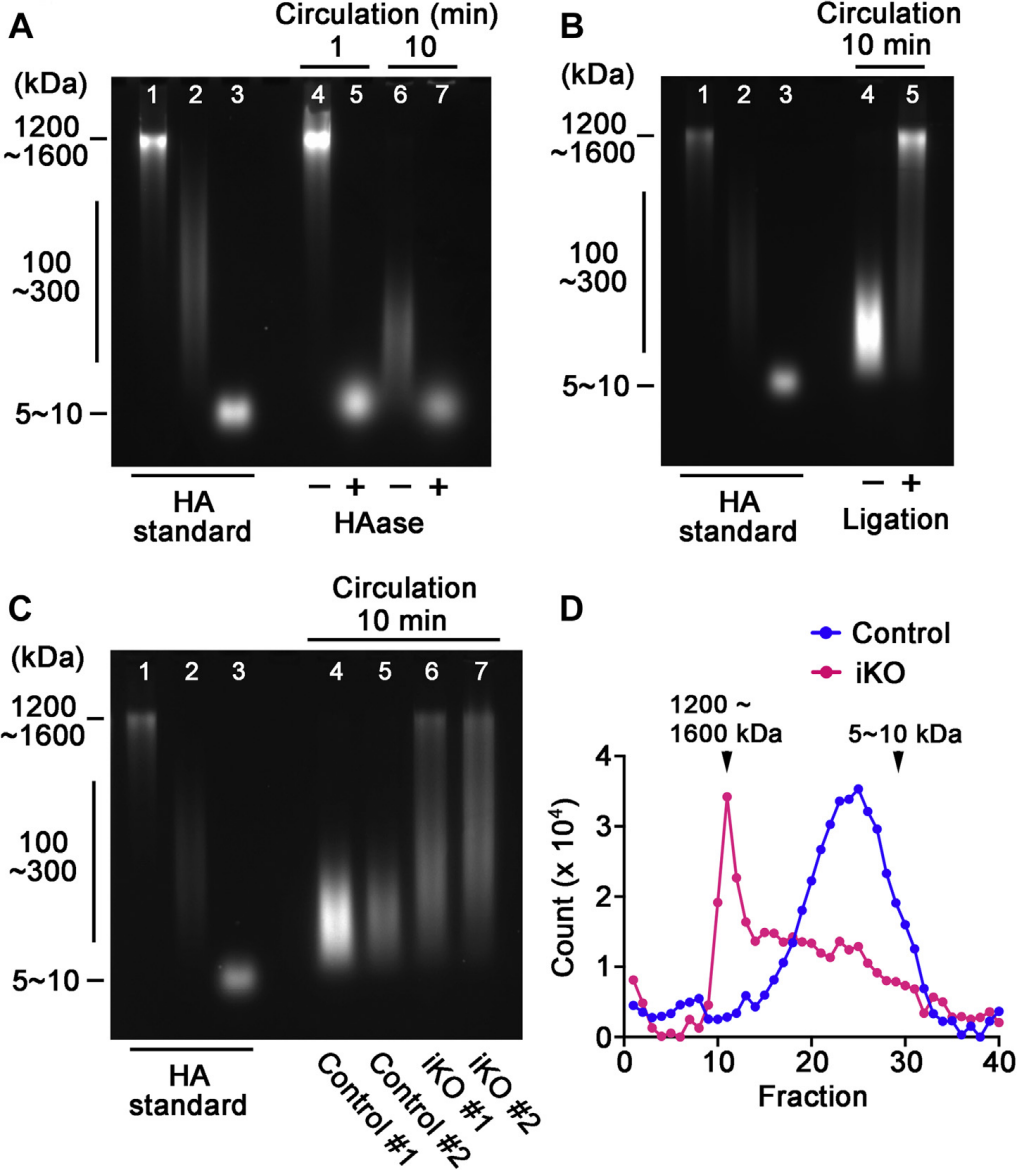
**Role of TMEM2 in dynamic HA degradation in the liver**. *A*, time course analysis of the degradation of circulating HA in wild-type mice. FA-HA (1200∼1600 kDa) was injected into IVC of adult wild-type mouse, and whole blood was collected from the heart at 1 or 10 min after injection. Degradation patterns of FA-HA in blood were analyzed by agarose-gel electrophoresis. *Lanes 1–3*: HA molecular weight standards (from left to right: 1200∼1600 kDa, 100∼300 kDa, 5∼10 kDa). *Lanes 4–5*: FA-HA in blood at 1 min after injection without (*lane 4*) and with (*lane 5*) hyaluronidase treatment. *Lanes 6–7*: FA-HA in blood at 10 min after injection without (*lane 6*) and with (*lane 7*) hyaluronidase treatment. *B*, Effects on FA-HA degradation of blocking blood flow into the liver. Immediately prior to FA-HA injection into IVC, blood flow into the liver was blocked by ligation of both the hepatic artery and the portal vein. FA-HA was injected into the IVC, and the experiments were performed with the 10 min interval as described above. *Lanes 1–3*: HA molecular weight standards (from left to right: 1200∼1600 kDa, 100∼300 kDa, 5∼10 kDa). *Lane 4*: FA-HA in blood without the blockade. *Lane 5*: FA-HA in blood with the blockade. Note that the blockade of the blood inflow markedly inhibits FA-HA degradation in blood. *C*, degradation pattern of IVC-injected FA-HA in circulating blood analyzed by agarose-gel electrophoresis. FA-HA injection experiments were performed without the blockade of blood circulation into the liver. *Lanes 1–3*: HA molecular weight standards (from *left* to *right*: 1200–1600 kDa, 100–300 kDa, 5–10 kDa); *Lanes 4–5*: FA-HA in control mice; *Lanes 6 to 7*: FA-HA in *Tmem2*^*iKO*^ mice. *D*, degradation pattern of IVC-injected FA-HA in circulating blood analyzed by gel filtration on Sepharose CL-2B. (*Red*) *Tmem2*^*iKO*^ mouse; control (*blue*) control mouse.

Using FA-HA as a tracer, we also examined the short-term dynamics of HA degradation in the liver, which is thought to be the primary site for degradation of HA in blood (1). For this analysis, we injected 50 μl of FA-HA (1200–1600 kDa; 0.2 μg/μl in PBS) into the inferior vena cava (IVC). Whole blood was then collected from the heart, and HA was analyzed by agarose-gel electrophoresis (Fig. 5, *A–C*) and gel filtration (Fig. 5*D*). As in the lymphatic FA-HA injection experiments, we first analyzed the time course of FA-HA degradation in wild-type C57Bl/6 mice. Little degradation of HA is observed at 1 min (Fig. 5*A*), while essentially all FA-HA is cleaved into intermediate-sized fragments after 10 min (Fig. 5*A*). We chose the 10 min postinjection interval for subsequent experiments. One complication with this analysis is that FA-HA injected into the IVC circulates not only to the liver but also to other organs such as the kidney and spleen that have been previously implicated in systemic HA catabolism. We therefore performed another pilot study to define the contribution of the liver to degradation of HA in blood. For this, immediately prior to FA-HA injection into IVC, the hepatic artery and the portal vein were ligated to block blood flow into the liver, and the FA-HA injection experiments were performed as described above. As shown in Figure 5*B*, degradation of FA-HA is almost entirely prevented by blocking circulation to the liver, demonstrating that our protocol provides a good approximation of liver-specific effects on the degradation of blood HA. Thus, we used the above FA-HA administration protocol (*i.e.*, a 10 min postinjection interval for blood collection and without ligation of the hepatic artery and portal vein) to compare the FA-HA degradation patterns between *Tmem2*^*iKO*^ and control mice. As shown in Figure 5*C*, liver-mediated degradation of FA-HA is greatly reduced in *Tmem2*^*iKO*^ mice in comparison with control mice. Analysis by gel filtration chromatography also demonstrates that in *Tmem2*^*iKO*^ mice, a significant portion of injected FA-HA remains undigested or only partially digested, whereas FA-HA is completely digested in control mice (Fig. 5*D*). These results demonstrate that TMEM2 in the liver plays a physiological role in the ongoing degradation of HA in the general circulation.

## Discussion

Prior to the identification of specific hyaluronidase molecules, systemic turnover of HA was studied quite extensively during the 1980s and 1990s, mostly by the group of Torvard Laurent at the University of Uppsala (1, 4, 10, 42, 43, 44, 45, 46, 47, 48). These studies revealed that: (i) HA is catabolized locally or carried by lymph to the lymph nodes and to the general circulation (1); (ii) most of the HA released from local tissues is degraded in lymph nodes, without reaching the circulation (45, 46); (iii) HA that reaches the general circulation is taken up and catabolized predominantly by the liver, while a minor portion is taken up by the kidney and the spleen (43); (iv) in the liver, sinusoidal endothelial cells are primarily responsible for HA uptake and degradation (49). Although the cell type responsible for HA uptake and degradation in the lymph nodes was not determined, lymphatic endothelial cells were suggested to be the responsible population (10); and (v) at the cellular level, internalized HA is transported to lysosomes and degraded to monosaccharides by the action of lysosomal hyaluronidase, β-glucuronidase, and β-*N*-acetylglucosaminidase (50). Subsequent to these pioneering studies, multiple candidate “hyaluronidase” molecules have been molecularly identified, and mutant mice deficient in these molecules have been generated. However, the respective roles of these hyaluronidase molecules in the specific steps of systemic HA catabolism remain poorly understood. This is at least partly because mice are not ideally suited for systemic HA metabolic studies due to limited size and accessibility of the blood and lymphatic vessels. Metabolic studies such as the ones cited above have generally used larger animals, such as rats and sheep, or human blood and lymph fluid specimens. As a result, studies of hyaluronidase knockout mice have relied mainly on HA accumulation in blood and tissues to deduce the functional significance of specific ablated hyaluronidase molecules. However, there are significant limitations associated with the use of HA accumulation as a surrogate parameter for evaluating HA degrading activity *in vivo*, because HA accumulation in blood and tissues is not a direct attribute of the loss of hyaluronidase activities. This limitation is further compounded when constitutive knockout mice are used for the analysis, since adaptive response compensating for the long-term loss of ablated hyaluronidase can complicate the interpretation of data and delineation of its physiological function.

To minimize these problems and to determine the metabolic role of TMEM2 in a more direct and selective manner, our current study employs a *CAG-Cre*^*ER*^-mediated inducible *Tmem2* knockout model. This model has allowed us to determine HA accumulation during a short window of time following the induction of *Tmem2* ablation. Furthermore, we have combined this inducible model with a tracer-based analysis of short-term HA degradation in order to directly analyze the effect of *Tmem2* ablation on ongoing HA catabolism. We find that within a few weeks of inducing *Tmem2* ablation, pronounced accumulation of HA occurs in blood and a variety of organs (Figs. 1 and 2, Tables S1, and S2). The HA population accumulated in blood contains a significant amount of undigested HMW HA species over 1000 kDa in size (Fig. 1*B*). Importantly, we show by direct analysis of FA-HA fragmentation that ongoing HA degradation is impaired in *Tmem2*^*iKO*^ mice in both the lymphatic system and the circulation (Figs. 4 and 5). We also demonstrate that *Tmem2* is predominantly expressed in endothelial cells in both lymph nodes and liver, consistent with the previous implication of these cell types in systemic HA catabolism (Fig. 3). Overall, these results support the aforementioned model for systemic HA metabolism and unambiguously demonstrate a physiologically relevant role for TMEM2 in systemic HA turnover.

The fact that TMEM2-mediated degradation of IVC-injected FA-HA requires less than 10 min (see Fig. 5) is consistent with a model in which TMEM2 functions on the cell surface (11). It is highly unlikely that FA-HA injected into the general circulation can be taken up and degraded intracellularly into fragments that are released back into the circulation within 10 min. Thus, our proposed model in which TMEM2-mediated HA degradation *in vitro* occurs on the cell surface (28) is likely also to be applicable *in vivo*. Overall, our data strongly suggest that TMEM2 expressed on the surface of liver sinusoidal endothelial cells plays a major role in the degradation of HA in the circulation. We surmise that TMEM2-mediated HA degradation in the lymphatic system operates in a similar manner on the surface of lymphatic endothelial cells.

Comparisons with published data from mouse knockouts of other hyaluronidase molecules provide useful insights into the relative importance of TMEM2 as physiological mediator of systemic HA turnover. Constitutive *Hyal1* knockout mice exhibit a modest 2-fold increase in serum HA (∼700 ng/ml), and there are essentially no effects on the size distribution of HA species (*i.e.*, HA exhibits essentially the same elution pattern in gel filtration analysis in both wild-type and knockout mice) (51). On the other hand, constitutive *Hyal2* knockout mice exhibit a 10–20-fold increase in blood HA (∼7000 ng/ml) (51, 52), accompanied by a shift in HA size distribution toward undigested HA species (51). Another study on *Hyal2* knockout mice in a mixed C129;CD1;C57Bl/6 background has reported a 19-fold increase in serum HA (53). Constitutive HYBID knockout mice have been examined in terms of their bone and nervous system phenotypes (54, 55). Although these mice have been reported to exhibit a 2.5-fold increase in the level of HA in hippocampal extracts, no analysis was performed for HA levels in blood or major organs. Thus, among these “hyaluronidase” molecules, HYAL2, like TMEM2, clearly makes substantial contribution to systemic HA catabolism. Nevertheless, it must be emphasized that the HYAL2 data were obtained from constitutive *Hyal2* knockout mice at the age of 4–7 months (51), and that the analysis did not include short-term *in vivo* degradation experiments of the type reported in our paper. Thus, while HYAL2 certainly participates in systemic HA catabolism in some manner, the data presented do not necessarily substantiate the claim that HYAL2 functions as a cell surface hyaluronidase (53, 56). This is because extracellular GAG accumulation can occur as a long-term consequence of defects in lysosomal GAG-degrading enzymes. For example, patients suffering from various mucopolysaccharidoses (MPS), including MPS type I (Hurler and Hurler–Scheie syndromes), MPS type II (Hunter syndrome), and MPS type III (Sanfilippo syndrome), exhibit widespread extracellular deposition of GAGs (57, 58, 59), even though the causal defects in these disorders reside in lysosomal enzymes. It is thus possible that the extracellular accumulation of HA observed in constitutive *Hyal2* knockout mice may not be the direct consequence of defects in cell surface HA degradation, but instead is caused by defects in lysosomal HS degradation.

The accumulation of HA in blood and organs in *Tmem2*^*iKO*^ mice is greater than the accumulation observed in constitutive knockouts of other hyaluronidase genes and occurs in a shorter time span following ablation of the gene (*e.g.*, 40.5-fold increase in circulating HA at just 19 days after the induction of *Tmem2* ablation; see Table S1). These results in *Tmem2*^*iKO*^ mice are even more remarkable considering the fact that, unlike the complete gene ablation achieved in constitutive knockouts, *CAG-Cre*^*ER*^-mediated gene ablation is by no means complete (31, 32, 33, 34, 35, 36, 37) (see Fig. S1*E*). The distinct significance of TMEM2 among the hyaluronidase molecules is also emphasized by the fact that both constitutive *Tmem2* knockout (*Tmem2*^*–/–*^) (Murao A., unpublished results) and neural crest-targeted *Tmem2* conditional knockout (*Wnt1-Cre;Tmem2*^*flox/flox*^) (60) mice are embryonic lethal and exhibit profound defects in multiple morphogenic processes. In contrast, none of the knockouts of other hyaluronidase genes (*Hyal1*, *Hyal2*, *Spam1*, *Hybid*) has lethal or significant embryonic phenotypes (41, 53, 54, 61, 62).

In conclusion, the data presented in this paper unambiguously demonstrate the significance of TMEM2 as a hyaluronidase molecule that plays an essential role in systemic HA catabolism *in vivo*. The experimental approaches employed in this study—*i.e.*, the use of an inducible global knockout system combined with short-term *in vivo* HA degradation analysis—can readily be applied to studies of other hyaluronidase molecules such as HYAL2 and HYBID for which loxP-modified conditional alleles have already been created. Comparison of metabolic phenotypes among these various hyaluronidase knockout models will provide critical insights into the relative importance and modes of action of different hyaluronidase molecules *in vivo*.

## Experimental procedures

### Generation of tamoxifen-induced Tmem2 knockout mice and analysis of Tmem2 expression

A conditional *Tmem2* null allele (*Tmem2*^*flox*^) was created by Cyagen Biosciences using TurboKnockout gene targeting methods. Mouse genomic fragments containing homology arms and the conditional knockout region were amplified from a BAC library and were sequentially assembled into a targeting vector together with recombination sites and selection markers. The targeting vector was electroporated into C57Bl/6-derived ES cells, followed by drug selection and isolation of drug-resistant clones. The resultant *Tmem2*^*flox*^ allele contains two loxP sites flanking exons 4 and 5 of the *Tmem2* gene, so that these two exons are deleted by Cre-mediated recombination (Fig. S1*A*). Exon 5 harbors amino acid residues, R265, D273, D286, mutagenesis of which individually abrogates the hyaluronidase activity of TMEM2 (28). Moreover, deletion of exons 4 and 5 results in conjugation of exon 3 to exon 6, creating a frameshift mutation and a premature stop codon (Fig. S1*D*). Tamoxifen-inducible, global *Tmem2* knockout mice were generated by crossing the *CAG-Cre*^*ER*^ transgene into *Tmem2*^*flox/flox*^ mice. *CAG-Cre*^*ER*^ transgenic mice (B6.Cg-Tg(CAG-cre/Esr1∗)5Amc/J; JAX mice 004682) were purchased from the Jackson Laboratory. Genotyping of mice and embryos was performed at weaning by PCR with the specific primers (Fig. S1, *B* and *C*) to analyze genomic DNA prepared from tail biopsies. *CAG-Cre*^*ER*^*;Tmem2*^*flox/flox*^, and *Tmem2*^*flox/flox*^ mice were treated with tamoxifen to generate *Tmem2*^*iKO*^ and control mice, respectively. For this, mice at 6 weeks of age were injected intraperitoneally with tamoxifen (0.125 mg/g body weight in corn oil; Sigma, T5648) for 5 consecutive days. At time points indicated in the text, whole blood was collected by cardiac puncture under anesthesia, followed by organs removal for the analysis of HA. Knockout efficiency in *Tmem2*^*iKO*^ mice was determined by TaqMan qPCR with total RNA isolated from the liver and lymph nodes. Liver and lymph nodes were dissected from *Tmem2*^*iKO*^ and control mice 3 days after the final tamoxifen injection and processed for isolation of total RNA using an NucleoSpin RNA Kit (Macherey-Nagel, 740955). cDNA was prepared from these templates using SuperScript VILO MasterMix (Thermo Fisher Scientific, 11755). *Tmem2* expression in the liver and lymph node was examined by using a LightCycler 96 system (Roche Applied Science) for TaqMan qPCR. Quantification was performed using a mouse *Tmem2* primer/carboxyfluorescein-conjugated probe, which spans exon 4 and 5 (Thermo Fisher Scientific, Mm01208442_g1). Levels of *Tmem2* expression were calculated *via* the ΔΔCt method with normalization to ribosomal 18S RNA (Thermo Fisher Scientific, Mm03928990_g1). Experiments were performed in biological triplicates or quadruplicates, and statistical significance was analyzed by Student's *t* test. All animal studies were performed in accordance with protocols approved by the IACUC of Sanford Burnham Prebys Medical Discovery Institute.

### Histological analyses

RNAscope ISH assays were performed using formalin-fixed paraffin-embedded sections according to the manufacturer's instructions. We used a commercial probe for mouse *Tmem2* (Mm-Tmem2; cat. # 496041) with RNAscope 2.5HD Reagent Kit-RED (Advanced Cell Diagnostics, 322350). Colorimetric images were captured using a Leica DM2500 light microscope. Some liver sections were double-labeled for *Tmem2* mRNA and CD31 protein. For this, sections processed for RNAscope ISH were subsequently incubated with anti-CD31 rabbit monoclonal antibody (clone D8V9E, Cell Signaling), followed by detection of CD31 with AlexaFluor 488-conjugated anti-rabbit antibody (Jackson ImmunoResearch, 711-545-152). Deposition of HA in tissues was examined by staining with a biotinylated HABP (hyaluronan binding protein) probe (AMSBIO, AMS.HKD-BC41) and with AlexaFluor 488-conjugated streptavidin (Thermo Fisher, S11223), as described previously (63). Some tissue sections were immunostained with rabbit polyclonal anti-laminin antibody (Sigma, L9393), followed by rhodamine red X-conjugated anti-rabbit IgG antibody (Jackson Immuno, 711-295-152). Fluorescence images were captured using a Zeiss LSM710 laser-scanning microscope.

### Analysis of lymphatic endothelial cells

Dissected peripheral lymph nodes were treated at 37 °C for 1 h with 1 mg/ml collagenase type 2 (Worthington, LS004176) in RPMI 1640 medium (Thermo Fisher Scientific, 61870036) containing 10% FBS (Corning, 35-010-CV). Dissociated cells were collected by passing through a 40 μm cell strainer, followed by centrifugation. Cell pellets were suspended in PBS containing 0.5% BSA and 2 mM EDTA and incubated on ice for 30 min with biotinylated anti-mouse CD31 antibody (clone: REA784, Miltenyi Biotec). Anti-biotin MicroBeads (130-090-485, Miltenyi Biotec) were then added to the cell suspension and incubated for an additional 15 min. CD31-positive cells were isolated using an MACS MS column (130-042-201, Miltenyi Biotec) according to the manufacturer's instructions. Total RNA was isolated from the CD31-positive and CD31-negative cell populations using an RNeasy Mini Kit (Qiagen, 74104), and cDNA was prepared by using SuperScript VILO MasterMix (Thermo Fisher Scientific, 11755). The TaqMan gene expression assay was carried out by using a LightCycler 96 system (Roche Applied Science) with a TaqMan primer/carboxyfluorescein-conjugated probe set for mouse *Tmem2* (Thermo Fisher Scientific; Mm00459599_m1). Absolute quantification of *Tmem2* mRNA copy numbers was performed as described previously (28) in biological triplicates, and statistical significance was analyzed by Student's *t* test.

### Quantification and biochemical analysis of HA

For the analysis of plasma HA, whole blood collected in a heparinized syringe was centrifuged at 1000*g* for 10 min. Supernatants were incubated with proteinase K (374 μg/ml; Roche, 03115828001) overnight at 55 °C. After inactivation of proteinase K at 95 °C for 10 min, the samples were further treated with DNase I (10 μg/ml; Sigma, DN25). For the analysis of tissue HA, tissues were digested with 200 μg/ml proteinase K for 12 h at 55 °C, followed by inactivation of proteinase K by heating the reaction mixtures at 95 °C for 10 min. HA content of these samples was measured using the Hyaluronic Acid LT Assay (Fujifilm Wako Chemicals) according to manufacturer's instructions. Total dsDNA in the samples was measured with Quant-iT PicoGreen dsDNA Assay Kit (Thermo Fisher, P7589). Tissue HA concentrations were calculated by normalization with respect to dsDNA concentration. The size distribution of plasma HA was analyzed by agarose-gel electrophoresis using HA standards of different average molecular weight (1000 kDa, Select-HA 1000k, Echelon Biosciences; 90–110 kDa, Sigma 75046; 8–15 kDa, Sigma 40583). Agarose-gel electrophoresis was carried out in 1% SeaKem agarose gels in TBE buffer for 30 min at 100 V, followed by staining with Stains-All dye (38). Specificity of staining for HA was confirmed by treatment of samples with *Streptomyces* hyaluronidase (1 unit/ml; Sigma H1136) in 100 mM sodium acetate buffer (pH 5.2) for 16 h at 37 °C.

### Analysis of *in vivo* HA degradation by the FA-HA tracer injection

*Tmem2*^*iKO*^ and control mice were used for these experiments 3 days after the final administration of tamoxifen (see above). Prior to both the lymph node and general circulation experiments, mice were anesthetized by intraperitoneal injection of Avertin (15 μl/g body weight). For HA degradation analysis in the lymphatic system, 15 μl of 100–300 kDa FA-HA (Cosmo Bio, CSR-FAHA-L2; 1 μg/μl in PBS) was injected subcutaneously into left hindpaw (for HA analysis in the popliteal lymph nodes) or forepaw (for HA analysis in the axillary lymph nodes) using a 50 μl Hamilton syringe fitted with a 30G needle. Six hours after injection, mice were euthanized, and the popliteal and axillary lymph nodes were removed. Lymph node tissues were incubated with 200 μg/ml proteinase K for 12 h at 55 °C. After digestion, proteinase K was inactivated by heating the reaction mixture at 95 °C for 10 min, and supernatants were collected by centrifugation (20,000*g* for 5 min).

For HA degradation analysis in the general circulation, laparotomy was performed on the mice and 50 μl of 1200–1600 kDa FA-HA (Cosmo Bio, CSR-FAHA-H2; 200 μg/ml in PBS) was injected into the IVC with an insulin syringe. Mice were exsanguinated either at 1 or 10 min after FA-HA injection by cardiac puncture using a heparinized syringe fitted with a 23G needle. Plasma (100 μl) from the whole blood sample was treated with proteinase K at 55 °C overnight to digest proteins, followed by the collection of supernatants by centrifugation (15,000 rpm on a Beckman Coulter Microfuge 20R for 5 min at 4 °C). From the supernatant (100 μl), HA was precipitated by adding 300 μl of 100% ethanol, followed by centrifugation at 15,000 rpm for 5 min at 4 °C. Precipitates were dissolved in 100 μl PBS. Patterns of FA-HA degradation were analyzed by agarose-gel electrophoresis and gel filtration. Agarose-gel electrophoresis was performed with FA-HA standards (Cosmo Bio, CSR-FAHA-H2, CSR-FAHA-L2, CSR-FAHA-U2) as described above, except that FA-HA was detected by a Bio-Rad ChemiDoc MP gel image analyzer. For size analysis by gel filtration, samples were applied onto a Sephacryl S-300 HR column (0.5 × 18 cm) or a Sepharose CL-2B column (0.5 × 18 cm) equilibrated in PBS. Fractions of 200 μl were collected at a flow rate of 0.2 ml/min, and fluorescence signals (excitation, 485 nm; emission, 535 nm) were measured on a CLARIOstar Plus multimode plate reader (BMG Labtech). For experiments using blockade of hepatic blood flow, laparotomy was performed as described above, and then each of the portal vein and the hepatic artery was quickly ligated at two sites with 5-0 branded silk sutures. Immediately after ligation, FA-HA was injected into the IVC, and the experiments were performed as described above.

### Data availability

All data are contained within the article and supporting information.

## Supporting information

This article contains supporting information.

## Conflict of interest

The authors declare that they have no conflicts of interest with the contents of this article.
