# Supplementary material for: The cell surface hyaluronidase TMEM2 is essential for systemic hyaluronan catabolism and turnover
Source: J Biol Chem. 2021 Oct 6;297(5):101281. doi: 10.1016/j.jbc.2021.101281 (PMC8561002; doi:10.1016/j.jbc.2021.101281)
Supplement: Table S1 [file mmc1.docx]

**Table S1. Quantity of plasma HA in control and *Tmem2^iKO^* mice.**

**HA (ng/ml plasma)**

**Days** **Control iKO fold *p* value**

12 176 ± 43 832 ± 63 4.7 0.0010

19 184 ± 53 7452 ± 1943 40.5 0.0132

Data represent nanograms of HA per milliliter of plasma at the indicated days after 5 days-tamoxifen injection. Values are shown as mean ± SEM from three to six biological samples. *p* value was determined by Student’s *t*-test.
