# Supplementary material for: The cell surface hyaluronidase TMEM2 is essential for systemic hyaluronan catabolism and turnover
Source: J Biol Chem. 2021 Oct 6;297(5):101281. doi: 10.1016/j.jbc.2021.101281 (PMC8561002; doi:10.1016/j.jbc.2021.101281)
Supplement: Table S2 [file mmc2.docx]

**Table S2. Quantity of tissue HA in control and *Tmem2^iKO^* mice.**

**HA (pg/ng DNA)**

**Tissue** **Control iKO fold *p* value**

Lymph node 210 ± 35 861 ± 86 4.1 0.0022

Liver 0.52 ± 0.08 4.32 ± 0.60 8.3 0.0012

Lung 762 ± 235 2615 ± 440 3.4 0.0208

Kidney 4548 ± 240 9879 ± 1733 2.2 0.0381

Data represent picograms of HA per nanogram of DNA at 19 days after 5 days-tamoxifen injection. Values are shown as mean ± SEM from three or four biological samples. *p* value was determined by Student’s *t*-test.
